# Racial/ethnic equity in substance use treatment research: the way forward

**DOI:** 10.1186/s13722-021-00256-4

**Published:** 2021-08-05

**Authors:** Kathleen Burlew, Caravella McCuistian, José Szapocznik

**Affiliations:** 1 Department of Psychology, University of Cincinnati, Mail Location #210375, Cincinnati, OH 45213 USA; 2 Philip R. Lee Institute for Health Policy Studies, University of California San Francisco, 3333 California St., Ste. 265, San Francisco, CA 94118 USA; 3 Department of Public Health Sciences, University of Miami Miller School of Medicine, 3380 Devon Road, Miami, FL 33133-5902 USA

**Keywords:** Racial/ethnic disparities, Racial/ethnic minorities, Drug treatment, Racial/ethnic investigator training, The National Drug Abuse Treatment Clinical Trials Network (CTN)

## Abstract

**Background:**

Opioid use and opioid-related overdose continue to rise among racial/ethnic minorities. Social determinants of health negatively impact these communities, possibly resulting in poorer treatment outcomes. Research is needed to investigate how to overcome the disproportionate and deleterious impact of social determinants of health on treatment entry, retention, drug use and related outcomes among racial/ethnic minorities. The current commentary provides recommendations that may help researchers respond more effectively to reducing health disparities in substance use treatment.

We begin with recommendations of best research practices (e.g., ensuring adequate recruitment of racial/ethnic minorities in research, central components of valid analysis, and adequate methods for assessing effect sizes for racial/ethnic minorities). Then, we propose that more NIDA research focuses on issues disproportionately affecting racial/ethnic minorities. Next, techniques for increasing the number of underrepresented racial/ethnic treatment researchers are suggested. We then recommend methods for infusing racial/ethnic expertise onto funding decision panels. This commentary ends with a case study that features NIDA’s National Drug Abuse Treatment Clinical Trials Network (CTN).

**Conclusions:**

The proposed recommendations can serve as guidelines for substance use research funders to promote research that has the potential to reduce racial/ethnic disparities in substance use treatment and to increase training opportunities for racial/ethnic minority researchers.

## Background

Although the opioid epidemic was initially most visible among non-Hispanic Whites, opioid use and overdose rates among other racial/ethnic groups (such as Blacks and Hispanics) have risen in recent years [1, 2]. These racial/ethnic minorities have worse treatment experiences and outcomes in substance use disorder (SUD) treatment than non-Hispanic Whites [1, 2]. For example, racial/ethnic minorities are likely to *enter treatment later* in their addiction process [3] and are *less likely to complete treatment* [4, 5]. Additionally, in an analysis of the 72,242 individuals in the Treatment Episode Datasets-Discharge (TEDS-D) dataset, *Blacks were less likely* to *reduce substance use than non-Hispanic Whites after treatment* [6]. Similarly, a meta-analysis by Windsor et al. (2015) found that Blacks in substance use treatment had *worse drug use outcomes* than non-Hispanic Whites [7].

The objective of this commentary is to suggest a way forward that maximizes the potential for conducting research aimed at reducing treatment outcome disparities in OUD and SUD among racial/ethnic minorities. The recommendations presented here include best research practices (e.g., recruitment of racial/ethnic minorities, central components of valid analyses, use of methodological techniques to support effect size comparisons), focusing research on urgent public health substance use treatment issues affecting racial/ethnic minorities, increasing the number of underrepresented racial/ethnic treatment researchers, and infusing decision-making on funding for treatment research with racial/ethnic minority investigators. The inclusion of racial/ethnic minorities will increase the likelihood that funded studies will generate knowledge about treatment outcomes for specific racial/ethnic groups.

After the recommendations, we present a case study that features NIDA’s National Drug Abuse Treatment Clinical Trials Network.

## Recommendations

### Recruit sufficient racial/ethnic minority participants

In 2017, the NIH released the Guidelines for the Inclusion of Women and Minorities as Subjects in Clinical Research [8]. Although a good first step, we question if these guidelines are sufficient. While we all believed that inclusion of women and minorities in clinical research was a useful step forward, once we determined the need to generate knowledge on what interventions work best with specific racial/ethnic groups, we recognized the limitations of this approach alone. We propose that investigators recruit a sufficient number of specific racial/ethnic groups to enable the study to generate some knowledge about the impact (or effect size) of the intervention on a specific racial/ethnic group. To do so, if including sufficiently large numbers of multiple racial/ethnic groups is not feasible, researchers might target a specific racial/ethnic group (e.g., one that experiences disparities related to one of the intervention outcomes). An adequate sample size for this group should be recruited to permit meaningful effect size analyses. For example, if the study aim is to reduce HIV risk among individuals who use substances, then targeting a racial/ethnic group with disparities in HIV rates may be more appropriate and feasible than including all racial/ethnic groups. To do so, investigators should consider the research aim early in the study design phase, examine the literature on the extent to which this research aim is relevant for specific target groups, and plan to over-sample for the specific racial/ethnic group(s).

### Central components of valid analysis

The 2017 amendment to those same *Guidelines for the Inclusion of Women and Minorities* attempted to define the valid analysis requirement. Although that amendment only stipulated separate analysis ‘by sex/gender and race/ethnicity’, we believe that conducting separate analyses for each race/ethnicity is only one step. Specifically, we propose the following as central components of valid analysis:

#### Assess for measurement equivalence

Recent scoping reviews of CTN research involving both Blacks [3] and Hispanics [9] revealed that measurement equivalence cannot be assumed across substance using racial/ethnic groups. Measurement equivalence refers to the extent to which a measure assesses the equivalent underlying psychological construct in a group other than the group in which the measure was standardized. Measurement equivalence is fundamental to the utilization of generic measures for assessing outcomes and other hypotheses [10] across racial/ethnic groups. Without measurement equivalence, researchers cannot be sure they are measuring the same construct across groups. Therefore, measurement equivalence must be established before comparisons can be made. In addition, limited representation of different racial/ethnic groups in the standardization of an instrument can impact generalizability of findings and have implications for racial/ethnic groups. For example, in our previous work on the Minnesota Multiphasic Personality Inventory (MMPI), we demonstrated that when a person’s scores were compared to his/her own racial/ethnic group, the interpretation differed from when his/her scores were compared against norms developed on a different racial/ethnic group [11].

#### Consider the caveats of race comparison designs

Race/ethnic group comparison designs may disregard the possibility that race/ethnicity may be a proxy for other socio-demographic differences (e.g., education, employment, income; 12). One particular concern is whether race/ethnic differences can be attributed to contextual factors (e.g., treatment barriers, enrollment in public vs. private clinics, neighborhood conditions) or the social determinants discussed below in recommendation 4. As mentioned earlier, measurement nonequivalence can lead to falsely indicating group differences. Moreover, given the heterogeneity of specific racial/ethnic groups, we encourage reviewers to focus more on within group differences rather than race comparison.

#### Consider within-race differences and the potential disadvantages of combining racial/ethnic groups in the analyses

It is critical to recognize the heterogeneity that exists within single racial/ethnic groups. For example, Weaver et al. (2015) found that rural Black women had lower odds of meeting criteria for Major Depressive Disorder than urban Black women [13]. Similarly, Guerrero et al (2013) noted that aspects of the cultural identity of specific subgroups of Hispanics (e.g., nationality, cultural values) rather than just general ethnic identity is more likely associated with substance use behaviors and would go unnoticed if Hispanic subgroups were analyzed together [14]. Furthermore, treating racial/ethnic groups as homogeneous groups often ignores the intersectionality of their race/ethnicity with other aspects of their identity such as gender, sexual orientation, educational status, etc. It is of even greater concern when investigators create a category called ‘minorities’ ignoring the vast heterogeneity within and across individuals of diverse racial/ethnic backgrounds. Heterogeneity within and across racial groups must be considered and adequately addressed.

### Utilize randomization methods that allow for assessing effect size for specific racial/ethnic groups

Reliance on traditional randomization methods may result in unequal assignment of racial/ethnic minorities across the treatment arms, precluding any rigorous comparison. Utilizing stratification methods that balance each race/ethnic group across treatment arms might render the data more suitable for calculating the effect size of an intervention for a specific racial/ethnic group. Both minimization [15] and blocked randomization [16] are potential strategies for balancing racial/ethnic group members in each treatment arm. While the numbers in each group may be insufficient to have adequate power for significance, effect sizes can be obtained to determine if the effect size is comparable to that obtained in the overall study, and for non-Hispanic Whites. If comparable, and if the intervention is efficacious/effective, then the study may provide pilot data to justify a larger study with a specific racial/ethnic group. If the effect size is much smaller for a specific racial/ethnic group, the findings may justify consideration of modifications (e.g., cultural adaptation) to increase the efficacy/effectiveness of the intervention for that specific racial/ethnic group. In this way, every randomized clinical trial can yield valuable information on whether the treatment has the potential to be efficacious/effective with a specific racial/ethnic minority group.

### Conduct research on treatment issues disproportionately affecting racial/ethnic minorities

Due to the identified disparities in treatment engagement, retention, and outcomes for racial/ethnic minorities, research addressing substance use treatment of racial/ethnic minorities is urgently needed. Since previous research has made a compelling case that social determinants of health are associated with racial/ethnic differences in substance use, researchers may begin with a literature review to determine which social determinants of health should be included for a specific racial/ethnic group.

When considering social determinants of health, we encourage researchers to consult Healthy People 2030, which outlines social determinants including education access and quality, health care and quality, neighborhood and built environment, social and community context, and economic stability [17]. These social determinants should be measured using rigorous assessment methods that have been standardized, ideally with the racial/ethnic groups of interest. Identifying standard ways to measure these social determinants of health across the NIH could support consistency and homogenization of data across projects. Additional harmonization could be achieved through approaches such as integrative data analysis [18] across multiple data sets.

Gaps also remain in research aimed at understanding unique issues related to treatment, such as interventions to overcome current barriers to early treatment entry, interventions to prevent lower treatment completion, and advantages of culturally adapted versions, including modifications to overcome any adverse impact of social determinants of health on substance use treatment outcomes. An example of this is outlined in a recent rapid review of opioid use in Black communities [19]. The authors posit that identifying social determinants of health and considering the role of culture in treatment are important steps for increasing treatment initiation and retention for this population. These authors suggest that both individual level and larger structural factors (e.g., location) are important to consider in future research.

### Expand the pipeline and increase racial/ethnic investigators’ access to research funding

Expanding the pipeline includes increasing access to training grants and research funding for racial/ethnic minority investigators. Racial/ethnic disparities exist in both. In 2017, among the NIH research training support for PhD student recipients, 66.3% (2,030) were non-Hispanic White but only 8.8% (266) were Hispanic and 5.4% (163) were Black [20].^1^ This reveals an overrepresentation of non-Hispanic Whites and an underrepresentation of Black and Hispanic students compared to the US population [21]. Moreover, Black and Hispanic investigators are less likely to receive funding than non-Hispanic White counterparts despite similarity in their educational background, training, and experience [22, 23]. In 2018, Blacks received 1.7% (214 of the 12,082) of the NIH research project grants. Similarly, Hispanics received 485 or only 4% of NIH research project grants [24].^2^ Although grant topic does not completely explain this underrepresentation, the NIH Deputy Director of Extramural Research (Michael Lauer) reported that grant applications by Black investigators are overrepresented in those institutes or centers (ICs) that fund the lowest number of awards [25]. In fact, of all the ICs, the National Institute on Minority Health and Health Disparities (NIMHD) has the lowest number of awarded R01 grants, which suggests that available funding for investigator-initiated research about racial/ethnic minority communities through NIMHD is extremely limited.

Several strategies may be useful for increasing the number of underrepresented racial/ethnic minority investigators. As suggested by NIH Director Dr. Francis Collins, structural racism contributes to the underrepresentation of racial/ethnic minorities in the biomedical workforce [26]. This in part occurs because biased social and historical conditions may have resulted in the lack of advancement in health sciences by racial/ethnic minorities. To remedy the impact of structural racism on diversity in the biomedical workforce, we propose two immediate mechanisms. First, we propose enhanced training to promising racial/ethnic minorities with the potential for independent research careers through the existing training mechanisms (e.g., F, K, T). Second, we propose that NIH support a significant number of underrepresented racial/ethnic minority young investigators through the minority research supplement mechanism which would enable them to join existing NIDA funded study teams.

### Infuse decision-making with racial/ethnic expertise

Racial/ethnic voices may bring a different but useful perspective to evaluating the value of a research proposal or may offer meaningful suggestions for modifying a research study proposal to increase its relevance for effectively addressing the substance use treatment needs of racial/ethnic minorities. The underrepresentation of racial/ethnic minorities on NIDA panels that evaluate research applications, perhaps inadvertently, prevents the field from reaping the benefit of hearing the voices of racial/ethnic minorities or other community representatives. Accordingly, we recommend expanding NIDA decision-making groups and research teams to include at least two underrepresented racial/ethnic group members immediately. Considering that underrepresented racial/ethnic group members comprise approximately a third of the US population [21], aiming for at least one fourth of each panel to be comprised of underrepresented racial/ethnic minorities in the next two fiscal years is a relatively modest bar.

### Case study: NIDA’s National Drug Abuse Treatment Clinical Trials Network (CTN)

The National Drug Abuse Treatment Clinical Trials Network (CTN) of the National Institute on Drug Abuse (NIDA) is a nationwide network of 15 university-based centers including treatment researchers, methodologists, data scientists, and community-based service providers who design, implement, and evaluate substance use treatment options for community-level clinical practice. Currently, the CTN is celebrating two decades of treatment research. Because approximately 43% of the participants recruited within CTN research protocols are racial/ethnic minorities, the CTN is uniquely positioned to address disparities in treatment research among racial/ethnic minorities.

The CTN has encountered a number of the issues discussed in this commentary. Members of the Minority Interest Group of the CTN and others have published several papers on addressing gaps in reaching and recruiting individuals from diverse groups into a study. These papers suggest that community engagement and the formation of academic/community research partnerships are both effective recruitment strategies [12, 27, 28]. Two recent scoping reviews of CTN research involving both Blacks [3] and Hispanics [9] revealed that measurement equivalence cannot be assumed across substance using racial/ethnic groups in CTN research. Moreover, several secondary analyses have revealed racial/ethnic differences in outcomes [3, 29–33]. Such findings are evidence of the need to investigate rather than to assume an intervention is equally effective across groups. Recently, the CTN, recognizing that the voice of racial/ethnic researchers was limited in study selection, added two racial/ethnic minority researchers to the committee that evaluates new study concepts. Moreover, the Minority Interest Group of the CTN is encouraging the CTN to expand the pipeline by increasing the diversity supplements associated with the CTN.

## Conclusion

The aforementioned recommendations comprise an initial statement of best practices in substance treatment research funding priorities, methods, training, and decision-making representation for addressing racial/ethnic disparities in OUD and other SUD treatment.

## Data Availability

Not applicable.

## Footnotes

1 These awards include: T15, T32, T90, TL1, TU2, F30, F31, F32, F33, F34, or F35.

2 These awards include DP1, DP2, DP3, DP4, DP5, P01, PN1, PM1, R00, R01, R03, R15, R21, R22, R23, R29, R33, R34, R35, R36, R37, R61, R50, R55, R56, RC1, RC2, RC3, RC4, RF1, RL1, RL2, RL9, RM1, UA5, UC1, UC2, UC3, UC4, UC7, UF1, UG3, UH2, UH3, UH5, UM1, UM2, U01, U19, and U34.
